# Mitochondrial Calcium Overload Drives mtDNA-cGAS-STING Activation via VDAC1 and MCU Upregulation in Periodontitis

**DOI:** 10.3390/ijms27104317

**Published:** 2026-05-12

**Authors:** Xinyi Cheng, Yu Cai, Yiran Geng, Xiaoying Zang, Jia Liu, Qingxian Luan

**Affiliations:** 1 Department of Periodontology, Peking University School and Hospital of Stomatology, National Center of Stomatology, National Clinical Research Center for Oral Diseases, National Engineering Research Center of Oral Biomaterials and Digital Medical Devices, Beijing 100081, China; cxy_perio@bjmu.edu.cn (X.C.); jessonjesson@hotmail.com (Y.C.); dentistgyr@126.com (Y.G.); 1910303134@pku.edu.cn (X.Z.); 2 Peking University Hospital of Stomatology Sanya Division (Sanya Stomatology Center), Sanya 572014, China

**Keywords:** periodontitis, calcium, mitochondria DNA, gingival fibroblasts, MCU, VDAC1

## Abstract

Periodontitis is a chronic inflammatory disease remaining elusive with its pathogenesis. Mitochondrial dysfunction and aberrant immune activation are implicated, but the underlying mechanisms remain incompletely understood. Given the essential role of Ca^2+^ homeostasis in maintaining normal mitochondrial function, we investigated the role of mitochondrial calcium (mtCa^2+^) dysregulation in periodontitis. Gingival tissues from periodontitis patients and healthy controls, as well as cultured gingival fibroblasts stimulated with *Porphyromonas gingivalis* lipopolysaccharide, were examined using transmission electron microscopy, confocal imaging, flow cytometry, qPCR, and western blotting. Notably, mtCa^2+^ was overloaded under inflammatory conditions, accompanied by disruption of whole-cell Ca^2+^ homeostasis. We also observed marked mitochondrial ultrastructural damage, mitochondrial DNA (mtDNA) leakage, and activation of the cyclic GMP-AMP synthase (cGAS)- stimulator of interferon genes (STING) pathway. The mitochondrial Ca^2+^ channel proteins, voltage dependent anion channel 1 (VDAC1) and mitochondrial calcium uniporter (MCU), were significantly upregulated in periodontitis gingiva, and their expression positively correlated with probing depth. Pharmacological inhibition of VDAC1 or MCU attenuated mtCa^2+^ overload, reduced mtDNA release and downregulated pro-inflammatory cytokines. These findings link mtCa^2+^ overload to mtDNA leakage and innate immune activation in periodontitis, and identify VDAC1 and MCU as promising therapeutic targets to restore mtCa^2+^ homeostasis and control host immune responses.

## 1. Introduction

Periodontitis is a chronic inflammatory disease of the tooth-supporting tissues. Severe cases lead to tooth loosening and loss, impairing masticatory function [1]. Current treatment focuses on mechanical debridement for plaque control, but some patients respond poorly and have high relapse rates, possibly due to aberrant host immune responses. The underlying mechanisms remain unclear, but these immune dysregulations may represent potential therapeutic targets as adjuncts to the conventional therapy [2].

Gingival fibroblasts (GFs) are the main cell type in gingiva, acting as immune sentinels, and preserving bone homeostasis [3]. GFs from periodontitis patients produce more reactive oxygen species (ROS) and higher Interleukin-6 (IL-6) and Interleukin-1β (IL-1β) levels than healthy controls [4]. In stage III/IV periodontitis, IL-6^+^ fibroblasts accumulated in residual pockets after non-surgical periodontal therapy. Coexistence of IL-6^+^ fibroblasts and Ficolin 1^+^ macrophages is significantly associated with osteoclast differentiation and bone resorption [5]. Collectively, GFs with a pro-inflammatory, high-oxidative-stress phenotype not only contribute to sustained inflammation but also cooperate with immune cells to regulate bone resorption homeostasis. Therefore, understanding the mechanisms driving this specific GF phenotype in severe periodontitis is urgently needed.

Mitochondria are a major source of ROS and also orchestrate innate immune responses. Upon failure to adapt to cellular stress, mitochondrial components such as mitochondrial DNA (mtDNA), mitochondrial RNA, and ATP are released into the cytoplasm, where they are recognized as pathogen-associated molecular patterns and activate immune responses–even cell death [6]. In periodontitis patients, gingival tissues or isolated GFs exhibit disrupted mitochondrial membrane structure, fractured cristae, upregulated mitochondrial ROS [7], and mtDNA leakage into the cytoplasm, which activates the cyclic GMP-AMP synthase (cGAS)-stimulator of interferon genes (STING) pathway [8] and PANoptosis [9]. Thus, the phenotype of GFs in periodontitis may be linked to mitochondrial dysfunction, and the triggers of this dysfunction require further investigation.

Mitochondrial Ca^2+^ (mtCa^2+^) homeostasis is essential for normal mitochondrial function [6]. Ca^2+^ promotes ATP production as a cofactor for oxidative phosphorylation, and mitochondria act as intracellular Ca^2+^ buffers. Physiologically, the cytoplasm, endoplasmic reticulum (ER), and mitochondria maintain dynamic Ca^2+^ balance: the cytoplasm maintains low Ca^2+^ concentrations, and even micromolar fluctuations can induce mitochondrial Ca^2+^ oscillations [10,11]. Low cytoplasmic Ca^2+^ activates ER inositol 1,4,5-trisphosphate receptors (IP3R), whereas high cytoplasmic Ca^2+^ inhibits them. The ER also directly transfers Ca^2+^ to mitochondria via mitochondria-associated membranes (MAMs) [12,13]. Therefore, mitochondrial Ca^2+^ plays a critical role in balancing intracellular Ca^2+^ distribution, but sustained mtCa^2+^ overload causes dysfunction: excessive ROS production and opening of the mitochondrial permeability transition pore (mPTP) [14], leading to damage-associated molecular patterns (DAMPs) leakage. In periodontitis, mtCa^2+^ overload has been observed, along with elevated MAM and mPTP levels [15,16]. However, the mechanisms underlying mtCa^2+^ dysregulation in periodontitis remain incompletely understood.

MtCa^2+^ influx is mediated by the outer-membrane voltage dependent anion channel (VDAC) and the inner-membrane mitochondrial calcium uniporter (MCU). In inflammatory diseases such as inflammatory bowel disease [17], cardiac ischemia-reperfusion [18], and Alzheimer’s disease [19], VDAC1 and MCU are upregulated. In inflammatory bowel disease mouse models, treatment with VBIT-12 ameliorates colonic epithelial injury, focal inflammation, colonic bleeding and serum mtDNA levels. In cardiac ischemia-reperfusion mouse models, MCU knockout or treatment with the natural MCU inhibitor berberine reduces cardiomyocyte oxidative stress and apoptosis, decreasing infarct size. The upregulation of VDAC1 and MCU in chronic inflammatory conditions may be relevant to trained innate immune memory. Palmitic acid increases H3K27ac enrichment at the *Vdac1* promoter in mouse hepatocytes [20]. MCU expression level correlates with STAT3 phosphorylation, which directly binds to the MCU promoter [21]. In the periodontal field, VDAC1 and MCU expression increases in lipopolysaccharide (LPS)-stimulated mouse bone marrow mesenchymal stromal cells and RAW264.7 cells, and *Porphyromonas gingivalis* induces VDAC1 oligomerization and mPTP opening in human aortic endothelial cells [22,23]. However, the relationship between VDAC1/MCU, Ca^2+^ homeostasis, and downstream inflammation in periodontitis remains poorly understood, and most evidence comes from cells or animal models without clinical validation. Therefore, further investigation into the roles of VDAC1 and MCU in mtCa^2+^ homeostasis and periodontal inflammation is warranted.

In this study, we verified that the hyperinflammatory phenotype of GFs is associated with sustained mtCa^2+^ overload and mtDNA leakage. We further examined VDAC1 and MCU expression in patients and animal models and demonstrated their regulatory role in inflammatory responses, thereby identifying VDAC1 and MCU as potential targets.

## 2. Results

### 2.1. Periodontitis Is Associated with Mitochondrial Dysfunction and mtDNA Leakage

**Figure 1:**
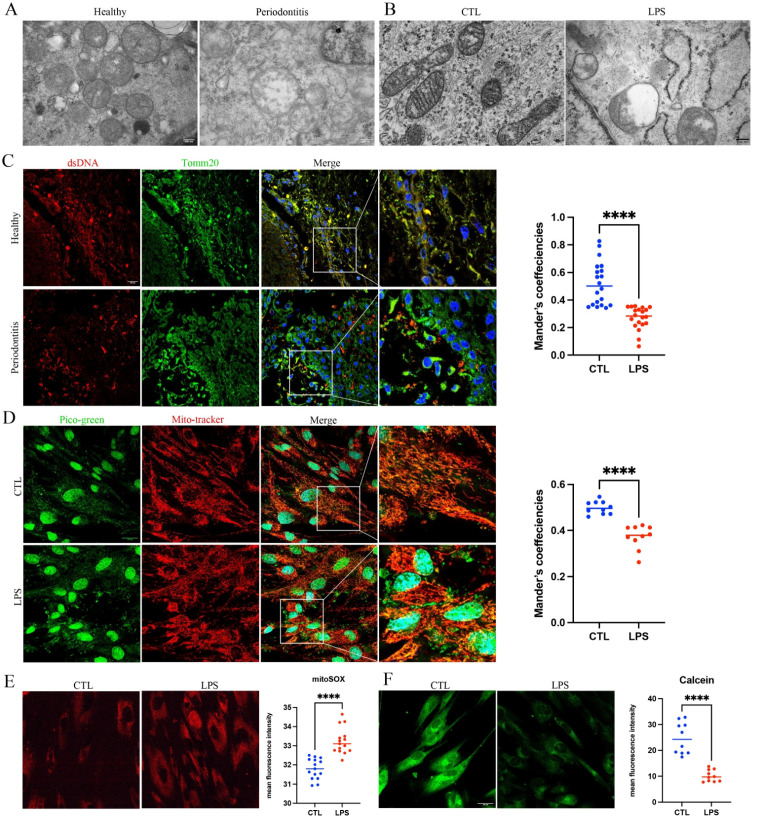
Structural and functional abnormalities of mitochondria in periodontitis tissues and cells. (**A**) Representative TEM images of human gingival tissue. Scale bar = 200 nm. (**B**) TEM images of healthy GFs with (LPS group) or without LPS (CTL group) stimulation for 24 h. (**C**) Immunofluorescence staining of human gingival tissue: dsDNA (red) labels double-stranded DNA, Tomm20 (green) labels mitochondria. Scale bar = 50 µm. Colocalization coefficients for dsDNA (nuclear DNA stained by DAPI excluded) and Tomm20 were quantified. (**D**) Representative fluorescence images of Pico-Green (green, dsDNA) and Mito-Tracker (red, mitochondria). Corresponding colocalization coefficients for Pico-Green (nuclear DNA stained by DAPI excluded) and Mito-Tracker are presented. Scale bar = 50 µm. (**E**,**F**) Representative images of GFs stained with MitoSOX and Calcein. Scale bar = 50 µm. Mean fluorescence intensities are quantified. **** *p* < 0.0001.

Transmission electron microscope (TEM) revealed that gingival tissues from periodontitis patients and GFs stimulated with LPS exhibited reduced electron density, disorganized and fragmented cristae, indicating ultrastructural abnormalities in mitochondria (Figure 1A,B). Consistent with these observations, mtDNA showed increased escape from mitochondria in periodontitis gingival samples (Figure 1C). Similarly, LPS-treated GFs displayed enhanced mtDNA leakage (Figure 1D). Accordingly, we found that LPS induced elevated mitochondrial ROS and mPTP opening in GFs (Figure 1E,F). These data indicate that periodontal inflammation is linked to mitochondrial dysfunction and mtDNA spillage.

### 2.2. Mitochondrial Calcium Overload Drives mtDNA Leakage

**Figure 2:**
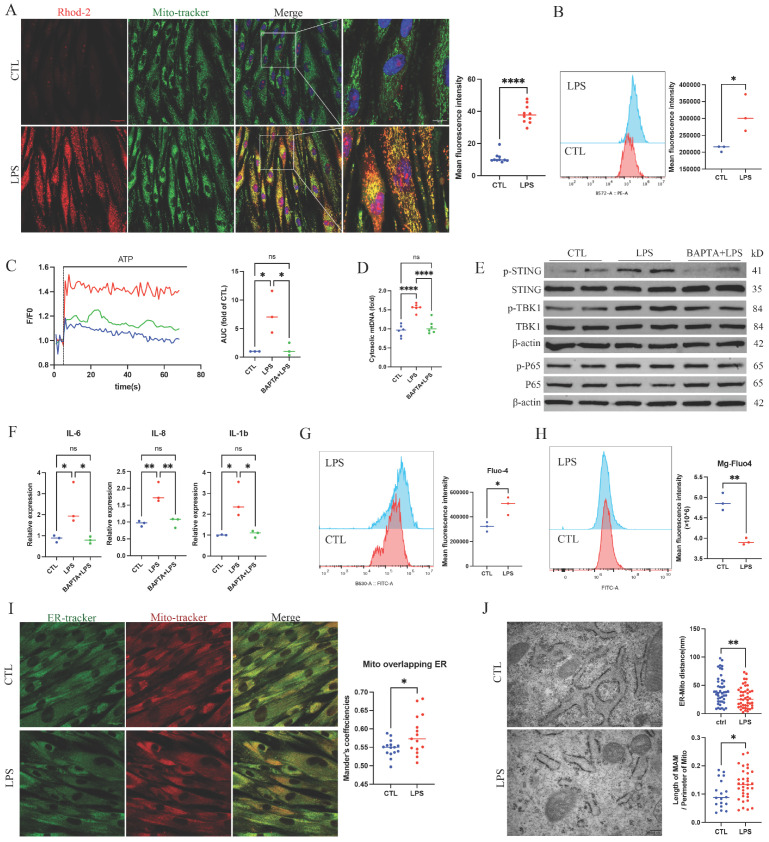
LPS stimulation induces Ca^2+^ dysregulation and activation of the mtDNA-cGAS-STING pathway in GFs. (**A**) Representative fluorescence images of mtCa^2+^ levels in GFs, with quantification of mean fluorescence intensity. Rhod-2 (red) indicates mitochondrial Ca^2+^ and Mito-Tracker (green) labels mitochondria. Scale bar = 50 µm. (**B**) Representative flow cytometry histograms and quantified mean fluorescence intensity of Rhod-2. (**C**) Representative flow cytometry time course of mitochondrial Ca^2+^ after ATP addition in healthy GFs pretreated with LPS (LPS group) or PBS (CTL group) for 24 h. BAPTA+LPS group received BAPTA (2μM) 2 h before LPS stimulation. Three independent replicates were performed and the AUC was calculated. (**D**) qPCR quantification of the ratio of cytoplasmic mtDNA to total mtDNA. (**E**,**F**) Western blot analysis of major cGAS-STING pathway proteins and mRNA expression levels of IL-6, IL-8, and IL-1β. (**G**,**H**) Representative flow cytometry histograms and mean fluorescence intensity of cytosolic Ca^2+^ (Fluo-4) and ER Ca^2+^ (Mg-Fluo-4). (**I**) Representative images of GFs stained with ER-Tracker (green) and Mito-Tracker (red), with quantification of colocalization coefficients. Scale bar = 50 µm. (**J**) Representative TEM images and quantification of ER-mitochondria distance (45 fields per group) and the proportion of mitochondrial perimeter in contact with ER (ER-mitochondria distance > 30nm excluded). Scale bar = 200 nm. ns, not significant, * *p* < 0.05, ** *p* < 0.01, **** *p* < 0.0001.

Previous studies [17,24] implicated mtCa^2+^ overload as a trigger for mtDNA release. At 24 h post-LPS stimulation, both fluorescence microscopy and flow cytometry revealed significantly elevated mtCa^2+^ levels in GFs compared with untreated controls (Figure 2A,B). To assess dynamic Ca^2+^ handling, ATP was added to LPS-treated GFs; ATP potentiates IP3R, an ER Ca^2+^ release channel, thereby rapidly increasing mtCa^2+^. The LPS-pretreated group showed a greater rise in mtCa^2+^ and slower decay than the control, indicating enhanced Ca^2+^ permeability and impaired homeostatic capacity. Quantification of the area under the curve (AUC) confirmed a significant increase of mtCa^2+^ in the LPS group (Figure 2C). Pre-treatment with the Ca^2+^ chelator BAPTA alleviated mtCa^2+^ overload, reduced mtDNA leakage, and attenuated downstream cGAS-STING activation and inflammatory cytokines upregulation (Figure 2D–F).

MtCa^2+^ is influenced by ER and cytosolic Ca^2+^ levels. Ca^2+^ transfer occurs not only indirectly through the cytosol but also directly via MAM. LPS treatment increased cytosolic Ca^2+^, decreased ER Ca^2+^ stores (Figure 2G,H), and upregulated MAM levels (Figure 2I,J). Collectively, these findings demonstrate that periodontal inflammation disrupts Ca^2+^ homeostasis, increases mitochondrial Ca^2+^ permeability, and activates the mtDNA-cGAS-STING pathway.

### 2.3. Mitochondrial Ca^2+^ Channel Proteins VDAC1 and MCU Are Upregulated in Periodontitis

**Figure 3:**
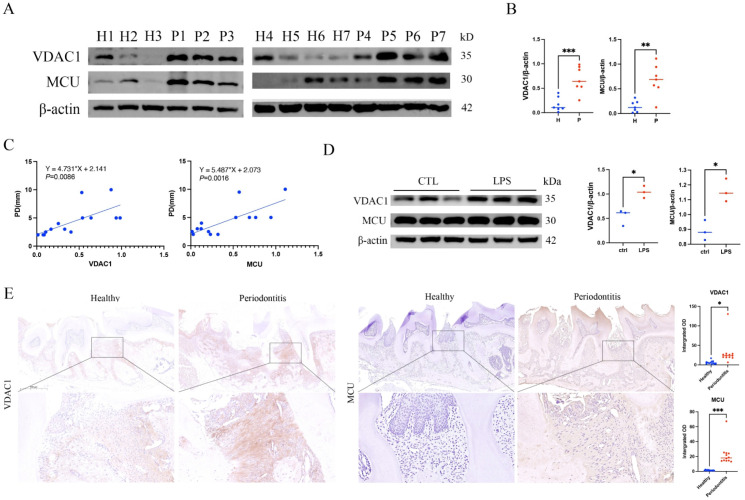
VDAC1 and MCU protein levels are upregulated in periodontitis tissues. (**A**,**B**) Western blot analysis of VDAC1 and MCU in gingival tissues from periodontitis patients (P) and healthy controls (H), with corresponding quantification. (**C**) Linear regression analysis of VDAC1 and MCU relative expression versus probing depth (PD) at the biopsy site. (**D**) Western blot analysis and quantification of VDAC1 and MCU in GFs. (**E**) Representative immunohistochemical staining of VDAC1 and MCU in mouse maxillae from the ligature-induced periodontitis model and healthy controls, with quantification of optical density. Red arrows, strong positive staining; black arrows, moderate staining; open arrowheads, very weak staining; solid arrowheads, negative staining. The red dashed line delineates the epithelium–connective tissue boundary. Scale bar = 500 µm. * *p* < 0.05, ** *p* < 0.01, *** *p* < 0.001.

To explore the mechanism underlying enhanced mtCa^2+^ permeability under periodontal infection, we examined the expression of mtCa^2+^-gating channel proteins. Gingival tissues from periodontitis patients (P) showed significantly higher VDAC1 and MCU protein levels than healthy controls (H), and their expression correlated positively with probing depth (PD) (Figure 3A–C, Table 1). In vitro, LPS-stimulated GFs also exhibited elevated VDAC1 and MCU protein levels compared with untreated cells (Figure 3D). VDAC1 and MCU expression were further assessed in a ligature-induced periodontitis mouse model. For VDAC1, the periodontitis group exhibited strong positive staining (red arrows) in the gingival epithelium, gingival fibrous connective tissue, and periodontal ligament, with overall expression markedly higher than that in the healthy group (black arrows). For MCU, the healthy group showed very weak staining in the gingival epithelium (open arrowheads) and essentially negative staining in the connective tissue (solid arrowheads); the periodontitis group displayed moderate positivity (black arrows), representing a clear increase relative to healthy controls (Figure 3E).

### 2.4. VDAC1 and MCU Are Potential Therapeutic Targets for Periodontitis

**Figure 4:**
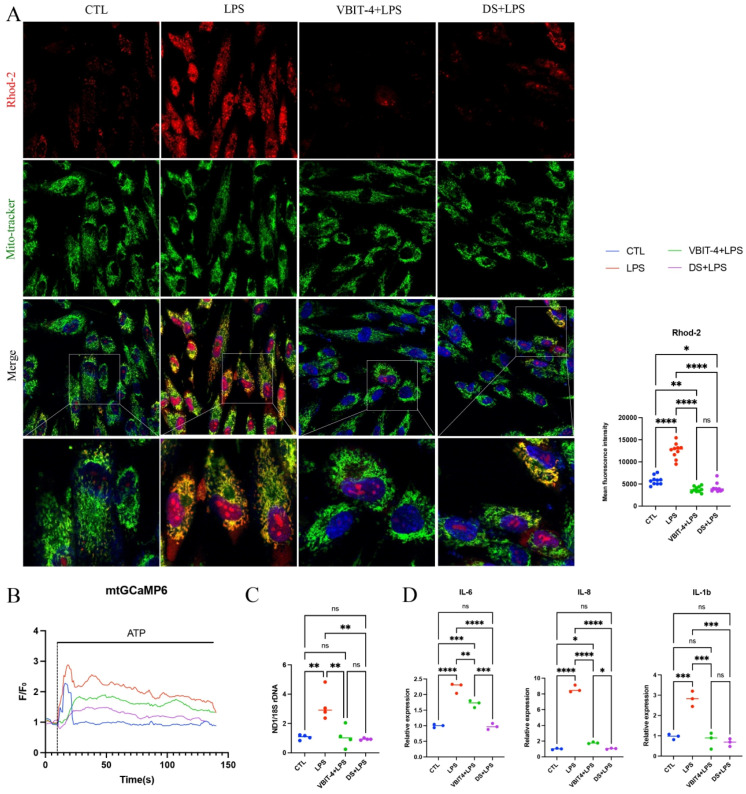
VBIT-4 and DS16570511 alleviate LPS-induced mitochondrial Ca^2+^ overload and mtDNA leakage. (**A**) Representative fluorescence images of mitochondrial Ca^2+^ (Rhod-2) in GFs pretreated with VBIT-4 (2 μM) or DS16570511(DS, 2 μM) for 2 h followed by LPS stimulation for 24 h, with quantification. VBIT-4+LPS and DS+LPS groups received inhibitor pretreatment before LPS; LPS and CTL groups received DMSO pretreatment followed by LPS or PBS control. Scale bar = 50 µm. (**B**) Representative flow cytometry traces of ATP-induced mitochondrial Ca^2+^ in mtGCaMP6-transfected GFs after treatments described in (**A**). Three independent replicates were performed and the AUC was calculated. (**C**) qPCR quantification of cytoplasmic mtDNA relative content and (**D**) Inflammatory cytokine expression levels in CTL, LPS, VBIT-4+LPS and MCU+LPS groups. ns, not significant, * *p* < 0.05, ** *p* < 0.01, *** *p* < 0.001, **** *p* < 0.0001.

To establish a causal link between VDAC1 and MCU and mtCa^2+^ overload, GFs were treated with VBIT-4 (the VDAC1 inhibitor) or DS16570511 (DS, the MCU inhibitor). Both pretreatments ameliorated LPS-induced mtCa^2+^ overload (Figure 4A). To more sensitively monitor mtCa^2+^, GFs were transfected with mtGCaMP6. VBIT-4 and DS each attenuated the sharp ATP-evoked rise in mitochondrial Ca^2+^ and improved mitochondrial Ca^2+^ permeability (Figure 4B). Regarding downstream immuno-inflammatory effects, both inhibitors reduced mtDNA leakage and suppressed inflammatory cytokine expression (Figure 4C,D). Together, these findings indicate that inhibiting VDAC1 or MCU modulates mtDNA-triggered innate immune responses, positioning them as promising therapeutic targets for periodontal inflammation.

## 3. Discussion

In this study, we observed mtDNA leakage and mtCa^2+^ overload in periodontitis and demonstrated that reducing excess mtCa^2+^ alleviates mtDNA release and cGAS-STING activation. We also found elevated expression of VDAC1 and MCU in periodontitis gingiva from patients and animal models, and blocking either channel reduced immune inflammation. These findings systematically link upregulated VDAC1 or MCU to aggravated mtCa^2+^ overload and subsequent mtDNA-driven inflammation.

Our data comprehensively reveal Ca^2+^ dysregulation in GFs under periodontitis: resting mtCa^2+^ is increased, dynamic Ca^2+^ handling is impaired; ER Ca^2+^ stores are depleted, and cytosolic Ca^2+^ rises. These findings align with the previous report of increased mtCa^2+^ and MAM in periodontitis gingiva and patient-derived periodontal ligament stem cells [15]. The mechanisms underlying this dysregulation remain incompletely understood but may involve a combination of cellular compensation and host inflammatory memory. Bacteria, oxidative stress products, ATP, etc. can activate channels on the plasma membrane [25] and ER [26,27], promoting Ca^2+^ influx into the cytosol and Ca^2+^ release from the ER. Mitochondria act as Ca^2+^ buffers. Upregulated VDAC1 and MCU, which are mitochondrial Ca^2+^ channels and integral components of MAM, enhance Ca^2+^ transfer from both the cytosol and the ER into mitochondria. However, sustained overload opens mPTP, allowing Ca^2+^ to flow back into the cytosol [10] while ATP production declines, impairing Ca^2+^ re-uptake into the ER and further depleting ER stores. We detected higher VDAC1 and MCU protein levels in periodontitis clinical sample, which were collected after scaling with relatively low bacterial load. The sustained upregulation of VDAC1 and MCU may reflect trained immunity following infection rather than active bacterial presence. Future studies using knockout or overexpression animal models are needed to test these hypotheses directly.

We found that reducing mtCa^2+^ overload decreased mtDNA leakage, cGAS-STING activation, and inflammatory cytokine expression. mtDNA is a prototypical mitochondrial DAMP implicated in various inflammatory diseases [28], including periodontitis. Jia Liu et al. [29] reported increased mtDNA leakage and mPTP opening in GF isolated from periodontitis patients, even in early passages. Xiang Liu [30] and Xue Jiang [31] demonstrated that cytosolic mtDNA activates either NOD-like receptor thermal protein domain associated protein 3 (NLRP3) or cGAS-STING in periodontitis. mtDNA can escape into the cytosol through at least four distinct pathways: mPTP opening, VDAC1 oligomerization, BAX/BAK oligomerization, and defective mitophagy. Sustained mtCa^2+^ overload directly triggers mPTP opening, allowing mtDNA to passively diffuse into the intermembrane space. Both VDAC1 and BAX/BAK oligomers are located on the outer mitochondrial membrane. VDAC1 is itself considered a component of the mPTP complex. NLRP3 inflammasome activator promotes ER Ca^2+^ release, which in turn elevates mtCa^2+^ and triggers VDAC1 oligomerization [32]. Jing Y et al. [33] proposed that mPTP opening occurs upstream and recruits BAX/BAK to promote their oligomerization. Whether Ca^2+^ directly regulates BAX/BAK oligomerization requires further investigation. In addition to these pore-forming pathways, excessive mtCa^2+^ and cytosolic Ca^2+^ suppress mitophagy, which together with Wnt/β-catenin pathway activation promotes osteoclast differentiation and accelerates periodontitis progression [34,35]. Collectively, these observations position Ca^2+^ as a central regulator of mtDNA release and subsequent innate immune activation in periodontitis.

This is the first report that VDAC1 and MCU are significantly upregulated in periodontitis gingiva and positively correlated with probing depth, which was also validated in cell and animal models. Pharmacological inhibition of VDAC1 or MCU ameliorated mtCa^2+^ overload, reduced mtDNA leakage, and suppressed downstream inflammation. However, their mechanisms likely differ. Inhibiting VDAC1 oligomerization not only alleviates mtCa^2+^ overload, thereby suppressing Ca^2+^-activated effectors such as calpain [36], but also directly blocks mtDNA release and suppresses NLRP3 inflammasome formation, achieving a more direct anti-inflammatory effect [32]. MCU is the only validated Ca^2+^ uniporter on the inner membrane, with low Ca^2+^ affinity [37]. DS16570511 inhibits MCU and MICU1, attenuating transient sharp Ca^2+^ spikes and thereby modulating the inflammatory cascade [38]. Furthermore, MCU downregulation may decrease VDAC1 dimerization and ubiquitination, preventing excessive mitophagy [39].

Limitations should be acknowledged. First, the clinical correlation analyses were based on a small sample (n = 7 per group). The significant associations between PD and VDAC1/MCU levels should be regarded as pilot findings and will need confirmation in larger cohorts. Second, evidence for mtCa^2+^ overload is currently limited to cell models and awaits direct validation in human gingival tissues. Third, this study assessed total MCU expression levels, without measuring the regulatory subunits (MICU1, MICU2, MCUb, and EMRE) or their stoichiometric ratios, which are essential for determining MCU Ca^2+^ permeability. Future studies should dissect the roles of specific subunits to enable more precise therapeutic targeting. Last, our study focused primarily on gingival fibroblasts, but periodontitis involves multiple cell types including macrophages, osteoclasts, and periodontal ligament cells. Whether VDAC1 and MCU play similar roles in these cell types remains to be investigated.

In summary, this study demonstrates that mtCa^2+^ homeostasis is essential for normal mitochondrial structure and function, limiting mtDNA leakage, and provides initial validation of VDAC1 and MCU as potential therapeutic targets for mitigating periodontal inflammation. Targeting these channels may offer a strategy to restore mitochondrial Ca^2+^ homeostasis and control host immune responses in periodontitis.

## 4. Materials and Methods

### 4.1. Human Clinical Samples and GFs Isolation

Fresh gingival samples were obtained from chronic periodontitis patients (n = 7) or periodontally healthy individuals (n = 7) undergoing crown lengthening or flap debridement. The study was approved by the Review Board and Bioethical Committee of Peking University Health Science Center (permit No. PKUS-SIRB-2013017). All subjects provided written informed consent in accordance with the 1975 Declaration of Helsinki. Demographic and clinical data are summarized in the Table 1. Samples for biochemical analysis were frozen in liquid nitrogen. For immunostaining, tissues were fixed in 10% formalin for 24 h and embedded in paraffin. For cell culture, gingival tissues were placed in Dulbecco’s modified eagle medium (DMEM) at 4 °C, and GFs were isolated within 1 h as previously described [4]. GFs at passages 3–6 were used. Cells were stimulated with 5 μg/mL *Porphyromonas gingivalis*-derived LPS (ATCC 33277, InvivoGen, San Diego, CA, USA), as detailed in our prior work [7].

### 4.2. Animals

Male C57BL/6J wild-type mice (6–8 weeks) were used for the periodontitis model and randomly assigned to periodontitis or healthy control groups (n = 6 per group). The experimental protocol was approved by the Institutional Animal Care and Use Committee of Peking University Health Science Center (protocol code DLASBD0510). Periodontitis was induced by ligating bilateral maxillary second molars with 5-0 silk sutures. Ligatures were checked every other day and re-ligated if loosened. After 2 weeks, maxillae were collected.

### 4.3. TEM

Gingival tissues and cells were fixed in 2.5% glutaraldehyde in 0.1 M phosphate buffer (pH 7.4) for 24 h, washed, post-fixed with 1% osmium tetroxide for 1 h at 25 °C, and rinsed. After dehydration in graded ethanol, samples were cleared in propylene oxide, infiltrated with Spurr’s resin, and polymerized at 60 °C for 48 h. Ultrathin sections were examined under a transmission electron microscope (JEOL, Akishima, Japan). Mitochondrial morphology and the ER-mitochondria distance (measure of MAM) were assessed.

### 4.4. Ca^2+^ Measurement

Mitochondrial, cytosolic, and ER Ca^2+^ levels were detected using Rhod-2 AM (2 µM, Beyotime, Shanghai, China), Fluo-4 AM (4 µM, Beyotime, Shanghai, China), and Mg-Fluo-4 AM (4 µM, Maokang Biotech, Shanghai, China), respectively. Cells were incubated with each probe at 37 °C for 30 min, washed three times with PBS, and incubated for another 30 min. Fluorescence was analyzed by confocal microscopy (Leica, Wetzlar, Germany) and flow cytometry (Agilent, Santa Clara, CA, USA). For Rhod-2 AM, baseline fluorescence was recorded for 20 s. ATP (MCE, Monmouth Junction, NJ, USA) was added to a final concentration of 1 mM (1/10 volume of the cell suspension), and measurement continued for 180 s. For GCaMP6s imaging, GFs were transfected with pcADV-CMV-4MT-GCaMP6s-3xFLAG (OBiO Technology, Shanghai, China) at the multiplicity of infection of 100:1 for 24 h, washed with PBS, and cultured in DMEM. Cells were then processed as above and analyzed by flow cytometry.

### 4.5. Mitochondrial Function Assays

Mitochondrial superoxide levels and mPTP opening were assessed using MitoSOX Red (Invitrogen, Carlsbad, CA, USA) and Calcein AM (Invitrogen, Carlsbad, CA, USA), respectively, following manufacturer protocols. ER and mitochondria were labeled with ER-tracker Green (Invitrogen, Carlsbad, CA, USA) and Mito-tracker Red (Invitrogen, Carlsbad, CA, USA). Fluorescence was visualized by confocal microscopy (Leica, Wetzlar, Germany). ER-mitochondria colocalization was quantified.

### 4.6. mtDNA Leakage Detection

GFs were harvested and divided into two aliquots. One aliquot was used for total DNA extraction (DNeasy Blood & Tissue Kit, Qiagen, Hilden, Germany). The other was permeabilized with digitonin (10 µg/mL, 10 min), centrifuged at 1000 rpm for 3 min, and the supernatant was further centrifuged at 17,000 *g* for 10 min at 4 °C to obtain the cytosolic fraction. Cytosolic DNA was extracted using the same DNeasy Blood & Tissue Kit. Mitochondrial ND1 in the total and cytosolic DNA were quantified by qPCR. Ratio of cytosolic mtDNA to total mtDNA was calculated. Primer sequences: ND1 (Forward: CACACTAGCAGAGACCAACCGAAC; Reverse: CGGCTATGAAGAATAGGGCGAAGG).

### 4.7. qRT-PCR

Total RNA was extracted with TRIzol (Thermo Fisher Scientific, Waltham, MA, USA), and cDNA was synthesized using ReverTra Ace qPCR RT Master Mix (Toyobo, Osaka, Japan). Real-time PCR was performed on a PikoReal 96 system with THUNDERBIRD SYBR qPCR Mix (Toyobo, Osaka, Japan) and gene-specific primers. Primer sequences were as follows:IL-6 (Forward: ATGAACTCCTTCTCCACAAGCGC; Reverse: GGGAAGGCAGCAGGCAACAC), Interleukin -8 (IL-8) (Forward: GCTCTGTGTGAAGGTGCAGTT; Reverse: TTTCTGTGTTGGCGCAGTGT), IL-1b (Forward: TGGCAACTGTTCCTG; Reverse: GGAAGCAGCCCTTCATCTTT), and glyceraldehyde-3-phosphate dehydrogenase (GAPDH) (Forward: GGAGCGAGATCCCTCCAAAAT; Reverse: GGCTGTTGTCATACTTCTCATGG).

### 4.8. WB

GFs (7.5×10^5^ cells per 6-cm dish) were treated with or without LPS. Cells and tissues were lysed in buffer containing 20 mM Tris-HCl (pH 7.5), 10% glycerol, 150 mM NaCl, 1% Triton X-100, 2 mM EDTA, and protease inhibitors (Roche, Basel, Switzerland). Protein concentration was determined by bicinchoninic acid assay. Samples were resolved on 10% SDS-PAGE, transferred to PVDF membranes, and blocked with 5% BSA in TBS. Membranes were probed overnight at 4 °C with antibodies: VDAC1 (sc-390996, Santa, Dallas, TX, USA), MCU (#14997, CST, Danvers, MA, USA), p-STING (#72971, CST, Danvers, MA, USA), STING (#13647, CST, Danvers, MA, USA), p-TBK1 (#5483, CST, Danvers, MA, USA), TBK1 (#3504, CST, Danvers, MA, USA), p-P65 (#3033, CST, Danvers, MA, USA), P65 (#8242, CST, Danvers, MA, USA), and β-actin (66009-1-Ig, Proteintech, Wuhan, China). After HRP-conjugated secondary antibody incubation, signals were visualized using a chemiluminescence kit (Thermo Fisher Scientific, Waltham, MA, USA).

### 4.9. Immunofluorescence and Immunohistochemistry

Paraffin sections (4 µm) were dewaxed in xylene, rehydrated in graded ethanol, and subjected to microwave antigen retrieval in 10 mM citrate buffer. Endogenous peroxidase activity was blocked with 3% H_2_O_2_ for 5 min, followed by blocking with 5% BSA for 1 h. Sections were incubated overnight at 4 °C with antibodies: VDAC1 (sc-390996, Santa, Dallas, TX, USA), MCU (HA723563, Huabio, Hangzhou, Zhejiang, China), dsDNA (sc-58749, Santa, Dallas, TX, USA), and Tomm20 (#42406, CST, Danvers, MA, USA), then with secondary antibodies. For immunohistochemistry, DAB was applied, followed by Mayer’s hematoxylin counterstaining, and images were captured under a light microscope (Olympus, Tokyo, Japan). For immunofluorescence, sections were counterstained with DAPI for 10 min and visualized using a confocal microscope (Leica, Wetzlar, Germany).

### 4.10. Statistical Analysis

Data were analyzed using GraphPad Prism 9.5 (GraphPad Software, San Diego, CA, USA) and presented as means ± standard deviation. Comparisons between two groups were made using Student’s *t*-test. Comparisons among multiple groups were made using one-way ANOVA followed by the Tukey post hoc test, or Brown-Forsythe and Welch ANOVA tests when standard deviations were not equal. Statistical significance was set at *p* < 0.05.

## Figures and Tables

**Table 1:** Clinical parameters at the surgery site of patients.

Group	Age	Gender	Smoking	GI	BI	PD	CAL	Teeth
H 1	57	Female	no	1	1	3 mm	0	13
H 2	32	Female	no	2	2	2 mm	0	25
H 3	45	Female	no	1	1	2 mm	0	12–13
H 4	59	Male	no	1	1	2.5 mm	0	13
H 5	35	Female	no	1	1	3 mm	0	16
H 6	37	Female	no	1	1	2 mm	0	46
H 7	43	Male	history	1	1	2.5 mm	0	12–13
P 1	55	Male	0.5 pack/day	2	4	10 mm	8 mm	28
P 2	43	Male	no	1	2	5 mm	1 mm	14
P 3	36	Male	no	2	3	5 mm	2 mm	21
P 4	26	Female	no	2	2	4 mm	1 mm	16
P 5	62	Female	no	2	2	5 mm	1 mm	27
P 6	32	Female	no	2	4	9.5 mm	7 mm	37
P 7	43	Female	no	1	1	5 mm	4 mm	14

H, Healthy control; P, Chronic periodontitis; GI, Gingival index; BI, Bleeding index; PD, Probing depth; CAL, Clinical attachment level.

## Data Availability

The raw data supporting the conclusions of this article will be made available by the authors on request.

## Abbreviations

The following abbreviations are used in this manuscript:

GF	Gingival fibroblast
LPS	Lipopolysaccharide
ROS	Reactive oxygen species
IL-6	Interleukin-6
IL-8	Interleukin-8
IL-1β	Interleukin-1β
mtDNA	Mitochondrial DNA
cGAS	Cyclic guanosine monophosphate-adenosine monophosphate synthase
STING	Stimulator of interferon genes
TBK1	TANK-Binding Kinase 1
mtCa^2+^	Mitochondrial Ca^2+^
ER	Endoplasmic reticulum
IP3R	1,4,5-trisphosphate receptors
MAM	Mitochondria-associated membranes
mPTP	Mitochondrial permeability transition pore
DAMPs	Damage-associated molecular patterns
VDAC	Voltage dependent anion channel
MCU	Mitochondrial calcium uniporter
TEM	Transmission Electron microscopy
qRT-PCR	Quantitative real-time polymerase chain reaction
WB	Western Blotting
SDS-PAGE	Sodium dodecyl sulfate polyAcrylamide gel electrophoresis
BSA	Bovine serum albumin
